# Analysis of Potential Circular RNAs in Regulating Imatinib Resistance of Gastrointestinal Stromal Tumor

**DOI:** 10.2174/1386207326666230822100024

**Published:** 2023-09-27

**Authors:** Jingyi Yan, Xiaolei Chen, Qiantong Dong, Ji Lin, Xuecheng Sun

**Affiliations:** 1 Departments of Gastroenterology, The First Affiliated Hospital of Wenzhou Medical University, Wenzhou, Zhejiang, 325000, China

**Keywords:** Circular RNAs, GIST, HIF-1, circular RNA array, imatinib mesylate secondary resistance, KEGG, gene ontology

## Abstract

**Introduction:**

Recent studies have found that circular RNA is an abundant RNA species that belongs to part of the competing endogenous RNA network (ceRNA), which was proven to play an important role in the development, diagnosis and progress of diseases. However, the function of circRNAs in imatinib resistance in Gastrointestinal stromal tumor (GIST) are poorly understood so for. The present study aimed to screen and predict the potential circRNAs in imatinib resistance of GIST using microarray analysis.

**Methods:**

We determined the expression of circular RNAs in paired normal gastric tissues (N), primary GIST (gastrointestinal stromal tumor) tissues (YC) and imatinib mesylate secondary resistance GIST tissues (C) with microarray and predicted 8677 dysregulated circular RNAs.

**Results:**

Compared with the YC group, we identified 15 circRNAs that were up-regulated and 8 circRNAs that were down-regulated in the C group. Gene ontology (GO) and Kyoto Encyclopedia of Genes and Genomes (KEGG) pathway analysis indicated that these host linear transcripts that differentially express circular RNAs are involved in many key biological pathways, predicting the potential tumor-genesis and drug resistance mechanismrelated to HIF-1 pathway, later we draw the cirRNA-miRNA-mRNA network involved in the HIF-1 pathway and found several dysregulated circRNAs and the relationship between circRNA-miRNAs-mRNA, such as circRNA_06551, circRNA_14668, circRNA_04497, circRNA_08683, circRNA_09923(Green, down-regulation) and circRNA_23636, circRNA_15734 (Red, up-regulation).

**Conclusion:**

Taken together, we identified a panel of dysregulated circRNAs that may be potential biomarkers even therapy relevant to the GIST, especially imatinib secondary resistance GIST.

## 1 INTRODUCTION

Gastrointestinal stromal tumor (GIST) is the most common gastrointestinal mesenchymal tumors [1]. The pathogenesis of GIST is mainly due to the protooncogene tyrosine kinase receptor KIT or PDGFRA-α gene activation mutation, as a result, abnormal activation of downstream signaling pathways, cell proliferation, apoptosis is inhibited and transformed into tumor cells [2, 3]. According to the type of gene mutation, GISTs were divided into KIT mutant (80%~85%), PDGFRA mutant (5%~10%) and wild type (10%) [4]. This provides a theoretical basis for the molecular targeted drug imatinib (IM) mesylate. IM is a drug that inhibits the activity of tyrosine kinase of KIT and PDGFRA gene, which is effective in the treatment of advanced GIST, and achieves satisfactory results [5]. However, more and more studies have found that IM occurs as primary and secondary resistance in the treatment process of GIST, and the mechanism of drug resistance is complicated. Thus, targeting KIT inhibitors alone does not benefit all GIST patients, especially in patients with wild-type GIST.

CircRNAs are a class of noncoding RNA (ncRNAs) molecules usually composed of more than one exon, formed mainly by back-splicing and covalent binding, which is misinterpreted as a rare event (splicing error) [6]. CircRNA functions as competitive endogenous RNA (ceRNA) efficiently targeting miRNA and inhibiting miRNA transcription like a molecular sponge, indirectly regulating mRNA expression [7]. CircRNA could regulate downstream gene expression through targeting miRNA and it may play an important effect in disease mechanisms. Inhibition of circ_0067934 could block metastasis, proliferation, and epithelial-mesenchymal transition (EMT) in non-small cell lung cancer (NSCLC) cells *via* miR-1182/KLF8 axis [8]. Regulation of Circ_0014130 could inhibit cell apoptosis in NSCLC cells by sponging miR-136-5p [9]. Over-expression of circ_0004015 could enhance resistance to gefitinib in NSCLC cells through the miR1183- PDK1 axis [10].

In this present study, we investigated the differentially expressed circRNAs using human circRNAs array in GIST tissues. We first demonstrated circRNAs of imatinib mesylate secondary resistance GIST and primary GIST, and found a correlation between circular RNA abundance and imatinib mesylate relapse resistance and make a function prediction.

## 2 MATERIALS AND METHODS

### 2.1 Patients and Clinical Specimen

The tumor tissues (all the tumor are greater than 5 cm) and matched normal gastric tissues were collected from 9 patients (three normal gastric tissue samples (N), three primary GIST samples (YC) and three GIST samples secondarily resistant to IM (C)) who underwent surgical resection at the First Affiliated Hospital of Wenzhou Medical University. All tissue samples were determined to be malignant GIST by pathology and immunohistochemistry (CD117(+), CD34(+), mitotic phase greater than 5/50HPF) and then stored in liquid nitrogen for further use. This study was approved by the Ethics Committee of the First Affiliated Hospital of Wenzhou Medical University and written informed consent was given before operation.

### 2.2 RNA Extraction, Library Construction and Sequencing

Total RNA was extracted using the mirVana miRNA Isolation Kit (Thermo) following the manufacturer’s protocol. RNA purity and quantification were evaluated using the NanoDrop2000 spectrophotometer (Thermo). RNA integrity was assessed using the Agilent 2100 Bioanalyzer (Agilent Technologies). Samples with RNA Integrity Number (RIN) ≥ 7 were used for subsequent library construction. After rRNA depleted and linear RNA digested by Ribonuclease R (Epicentr), library construction using TruSeq total RNA and Ribo-Zero Gold (Illumina). Then, we sequenced these libraries on the Illumina sequencing platform (HiSeq X Ten) and 150 bp paired-end reads were generated. circular RNA sequencing analysis and conducted by OE Biotech Co., Ltd. (Shanghai).

### 2.3 Bioinformatic Analysis

Raw data (raw reads) of fastq format were first processed using the Trimmomatic software [11]. Clean data (clean reads) were obtained by removing reads containing adapter, reads containing ploy-N and lower quality reads from raw data. Clean reads were aligned to the reference genome GRCh38 utilizing the MEM algorithm of Burrows-Wheeler aligner (BWA, version0.7.5a) [12]. Based on the junction reads and GT-AG splicing signals, circRNAs were verified using CIRI2 software [13]. Combined with annotation information in the protein database, circRNAs were annotated for further analysis. RPM determined circRNAs level. To identify differentially expressed circRNAs, statistical comparison between two different groups was determined by the DESeq (2012) R package [14], with setting the threshold of adjusted p-value < 0.05 and foldchange > 2 or foldchange < 0.5. FDR (false discovery rate) is used as the p-value threshold for multiple tests to judge the significance of gene expression differences. The interaction of circRNA-miRNA was predicted by miRanda software with the threshold of score > 150, energy < -30 and strict paired in the seed region [15]. The target genes of miRNA were predicted based on the intersection of the results on miRWalk and miRDB database. Differentially expressed gene data of the group C *vs* YC were obtained from another research [16].

### 2.4 Gene Function Analysis

GO terms and Kyoto Encyclopedia of Genes and Genomes (KEGG) pathway enrichment analysis were used to predicate the functions of circRNAs [17, 18]. KEGG analysis was performed to determine the involvement of target genes in different biological pathways using KOBAS software [19].

### 2.5 Construction of circRNA-miRNA-mRNA Interaction Networks

miRanda software was used to predict the interaction of circRNA-miRNA with the threshold of score > 150, energy < -30 and strict paired in the seed region. The target genes of miRNA were predicted based on the intersection of the results on miRWalk and miRDB database. CircRNA-miRNA-mRNA network was visualized by the Cytoscape software.

## 3 RESULTS

### 3.1 C-KIT Two Mutation Sites in Patients with Imatinib Mesylate Secondary Resistance

**Fig. (1):**
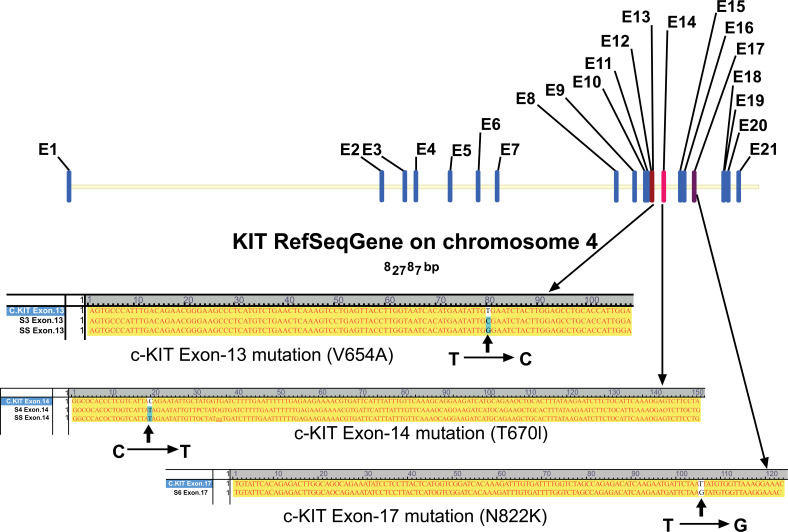
C-KIT two mutation sites in patients with imatinib mesylate secondary resistance. C-KIT secondary mutation sites in patients with imatinib mesylate secondary resistance GIST (one case locus in the v654a of exon 13, one case locus in the T760I of exon 14, another one locus in the v654a of exon 17).

All specimens were analyzed by RT-PCR amplification, DNA sequencing and analysis and then comparison with wild type c-KIT gene, C-KIT/PDGFR-α mutation were detected, secondary mutations were found in 3 GIST specimens with drug resistance. One case locus was observed in the v654a of exon 13, another case locus in the T760I of exon 14, and the other locus in the N822K of exon 17 (Fig. **1**).

### 3.2 Identification and Characteristics of circRNAs

**Fig. (2):**
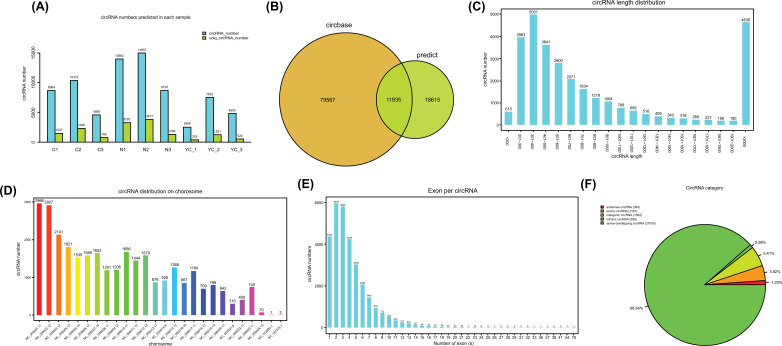
Identification and characteristics of circRNAs. (**A**) Identification of circRNAs in different samples. (**B**) Venn analysis for comparison of predicted circRNAs with the data published in the circBase. (**C**) The length distribution of circRNAs. (**D**) Chromosome distribution of circRNAs. (**E**). Distribution of the exon numbers of circRNAs. (**F**). Category of circRNAs based on genomic origin.

To explore circRNA expression profiles in N, Y or YC, and C group, we performed ribosomal RNA-depleted RNA sequencing and the number of circRNAs identified in each sample is shown in Fig. (**2A**). Venn analysis showed that 11935 circRNAs were found between predicted circRNAs and circBase (Fig. **2B**). According to the circular RNAs array, a total of 30,550 were detected in 9 samples, and the length mostly distribute in 201-400bp and >2000bp (Fig. **2C**). Chr1, Chr2 and Chr3 are the three most located chromosomes (Fig. **2D**). Most circular RNAs have less than 6 exons (Fig. **2E**). Similarly, most of the identified circular RNAs (27050, 88.54%) came from overlapping regions of meaning, indicating that the formation of circular RNAs is closely related to the pre-mRNA splicing mechanism (Fig. **2F**). Approximately 3.82% (1167) and 5.41% (1652) circular RNAs were derived from exons and intergenic regions. A small part of circular RNA is antisense circular RNA (383, 1.25%) and intronic circRNAs (298, 0.98%). General features of the circRNA sequencing data were list in Table **1**.

### 3.3 The Potential Functions Identification

**Fig. (3):**
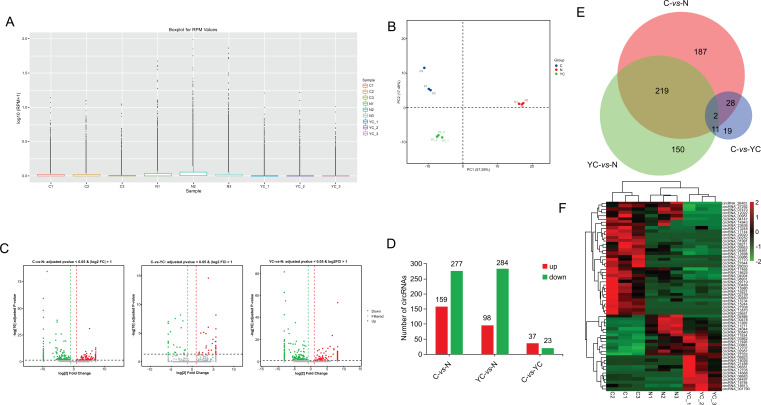
Differentially expressed circRNAs in the N, C and YC groups. (**A**) Box plots of reads per million (RPM) values of circRNAs in each sample. (**B**) PCA plot of all the samples in N, C and YC groups. (**C**) Volcano plots indicated the variation of circRNA expression in different comparison groups C *vs* N, YC *vs* N, C-vs-YC. (**D**) Column chart of differentially expressed circRNAs in each comparison. The numbers on column show the numbers of up-regulated (red) and down-regulated (green) circRNAs. (**E**) Venn analysis of the three comparison groups. (**F**) Heatmap of differentially expressed circRNAs between YC and C groups.

A recent study has reported that circRNAs possess has tissue-specific expression characteristics. We used DEseq software to analyze circRNA expression profiles RPM to screen dysregulated circRNAs in three different GIST samples, and found that that no abnormal expression was observed in three different GIST samples (Fig. **3A**). PCA (Principal Component Analysis) was performed to analyze the circRNA expression profiles of the three group's samples. The distance between points represented the similarity between the two samples, and the repeatability of the three groups of samples was ideal in Fig. (**3B**). Differential analysis was conducted among the three comparison groups by Volcano plots. The circRNA differentially expressed was screened using the criteria of “adjusted pvalue < 0.05 and absolute value of log2Foldchange >1”. The red dot on the volcano map significantly increased circRNA, the green dot significantly reduced circRNA, and the gray dot showed no obvious difference (Fig. **3C**). These were 159 circRNAs up-regulated in comparison C-vs-N group; 98 circRNAs up-regulated in comparison YC-vs-N group; and 37 circRNAs up-regulated in comparison C-vs-YC group; 277 circRNAs down-regulated in comparison C-vs-N group; 284 circRNAs down-regulated in comparison YC-vs-N group, and 23 circRNAs down-regulated in comparison C-vs-YC group (Fig. **3D**). Venn analysis of the three comparison groups was shown in Fig. (**3E**). In general, the same kind of samples can be clustered in the same cluster, and the genes in the same cluster may have similar biological functions, our results show that all samples in paired groups have the co-regulated (up or down) genes (Fig. **3F**). The top ten different expression circRNA in the three comparison groups was shown in Table **2**.

### 3.4 GO Enrichment Analysis for the Host Genes of Differentially Expressed circRNAs

**Fig. (4):**
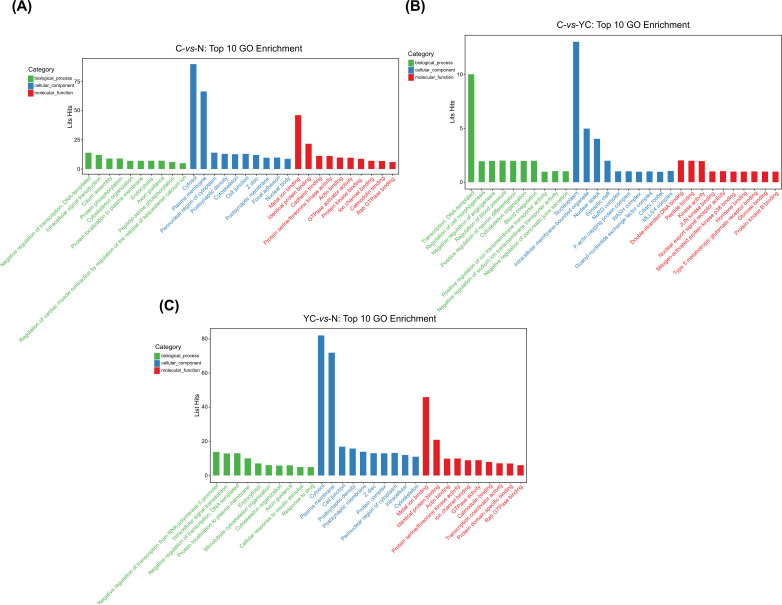
GO enrichment analysis for the host genes of differentially expressed circRNAs. (**A-C**). GO enrichment analysis was conducted based on the host genes of differentially expressed circRNAs. The bar diagrams show the top10 enriched GO terms (*P*<0.05) in the group C *vs* N (**A**), YC *vs.* N (**B**), C-*vs*-YC (**C**), respectively.

After obtaining the differentially expressed genes, we selected the top10 functional enrichment analysis. The enriched functional terms were used as the predicted functional term of given circRNAs. Analysis of the difference gene expression with GO analysis was used to describe its function (with GO annotation). GO analyses covered three subgroups: biological process (BP), cellular component (CC), and molecular function (MF). The GO analysis with the most significant enrichment in the BP, CC, and MF subgroups by C-vs-N comparison groups is the regulation of transcription, DNA-templated, cytosol and metal ion binding, respectively. In C-vs-YC group, the GO analysis with the most significant enrichment in the BP, CC, and MF subgroups is the regulation of transcription, DNA-templated, nucleoplasm and double-stranded DNA binding. In YC-vs-N group, the GO analysis with the most significant enrichment in the BP, CC, and MF subgroups is the regulation of transcription from RNA polymerase II promoter, cytosol and metal ion binding (Fig. **4A-C**).

### 3.5 Construction of the circRNA-miRNA Interaction Network in Drug Resistance/HIF-1

**Fig. (5):**
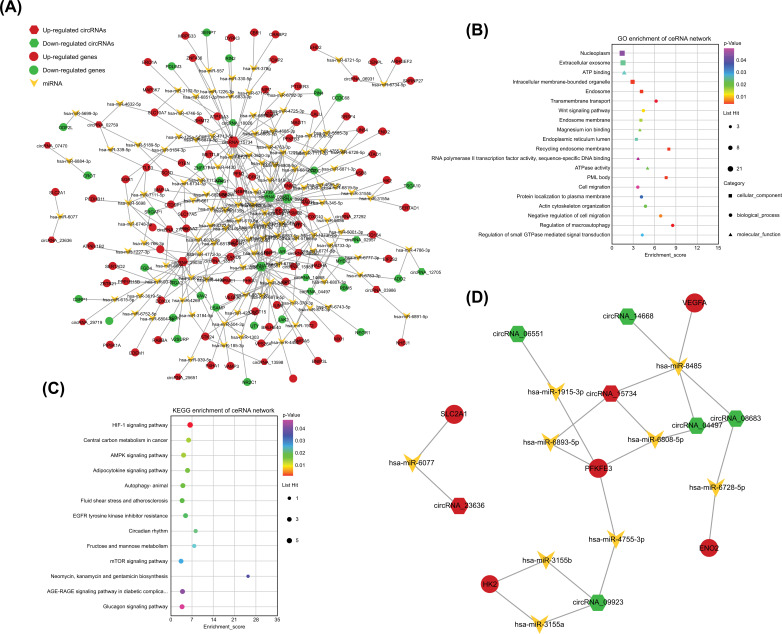
The ceRNA regulatory network in the group C *vs.* YC. (**A**) the cirRNA-miRNA-mRNA network in the group C *vs.* YC. Red, up-regulated. Green, down-regulated. Hexagon, circRNAs. Circle, mRNA. Arrowhead, miRNA. (**B**) GO enrichment analysis of the cirRNA-miRNA-mRNA network. The bubble diagram shows the top 20 enriched GO terms (*P*<0.05). (**C**) KEGG pathway enrichment analysis of the cirRNA-miRNA-mRNA network. The bubble diagram shows the enriched pathways (*P*<0.05). (**D**) The cirRNA-miRNA-mRNA network involved in the HIF-1 pathway.

We combined the chip data (OE2016Q1031Y) from another of our published articles to draw the cirRNA-miRNA-mRNA network in the group C *vs.* YC, and found that 15 cirRNAs were up-regulated (Red) and 8 cirRNAs were down-regulated (Green) (Fig. **5A**). GO enrichment analysis of the cirRNA-miRNA-mRNA network, the bubble diagram shows the top 20 enriched GO terms (*P*<0.05) (Fig. **5B**). KEGG pathway enrichment analysis of the cirRNA-miRNA-mRNA network, and found out the potential relationship between differential expression genes with changes in cell pathways, such as HIF-1 pathway, Central carbon metabolism in cancer, AMPK signaling pathway, Autophagy-animal and so on (Fig. **5C**). Later, we analyzed the cirRNA-miRNA-mRNA network involved in the HIF-1 pathway and found that the correlation between each dysregulated circRNA-miRNAs-mRNA, circRNA_06551, circRNA_14668, circRNA_04497, circRNA_08683, circRNA_09923(Green, down-regulation) and circRNA_23636, circRNA_15734(Red, up-regulation) (Fig. **5D**).

## 4 DISCUSSION

CircRNAs have a covalently closed loop structure without 5’and 3’termini, mainly caused by back-splicing and covalently binding, which was detected 20 years ago but considered a rare event for so long [20, 21]. Recent studies have proved that circRNAs play critical roles in the development and prognosis of many diseases, such as Alzheimer’s, cardiac hypertrophy, heart failure, and cancers [22]. Now circRNAs are thought to be a promising direction in a variety of diseases. Various studies have shown that circRNA is deregulated in different types of human cancers [23-26]. CircRNA plays a vital effect in the biological processes involved in tumor progression and drug resistance [27]. It also acts as a microRNA (miRNA) sponge and RNA-binding protein sponge, for gene transcription. For instance, over-expression of circRNA_0025202 could regulate tamoxifen sensitivity through regulation of the miR-182-5p/FOXO3a axis in breast cancer [28]. Circular RNA AKT3 could regulate drug resistance in gastric cancer (GC) cells *via* inhibition of miR-198 and upregulation of PIK3R1 [29]. Up-regulation of Circular RNA MCTP2 could inhibit resistance to cisplatin in GC by regulation of miR-99a-5p/ MTMR3 axis [30]. Inhibition of circCELSR1 could enhance sensitivity to paclitaxel in ovarian cancer cells vis FOXR2 /miR-1252 axis [31]. However, the expression profiles and functions of circRNA in GIST IM resistance are still unclear.

Supporting the theory that circRNAs may be a new and stable biomarker and breakthrough therapeutic direction for now-known intractable diseases. In the present study, we explored the circRNA expression patterns in 9 patients N, Y or YC and C group using high-throughput RNA sequencing. Obtained microarray specimen results show that most of the circRNAs are about 201-400bp in length, which is consistent with the previous report that the median length of circRNA is about 500 nt [32]. Circular RNA is mainly produced by the exons or introns of its host linear transcript and participates in the regulation of host gene expression [33, 34]. Therefore, following screening the differentially expressed circRNAs between Y and C tissue samples, we used GO and KEGG pathway analyses to predict the biological functions of their host linear transcripts, indicating that it was involved in the HIF-1 signaling pathway.

HIF is often up-regulated in different cancer cells and is related to the progression and poor clinical outcomes of many tumor entities [35, 36]. HIF genes regulate the expression of many genes related to angiogenesis, tumor growth, metastasis, and therapeutic resistance [37]. Hypoxia-inducible transcription factor 1α (HIF-1α), was identified as the main regulator of hypoxia-induced drug resistance and is considered to be an attractive target for tumor therapy [38]. The present study constructed the cirRNA-miRNA-mRNA network involved in the HIF-1 pathway through bioinformatic prediction and clarified cirRNA-miRNA-mRNA axis participation in this regulatory network. We found several circRNA being up-regulated or down-regulated, such as circRNA_23636, circRNA_15734 (up-regulation); circRNA_06551, circRNA_14668, circRNA_04497, circRNA_08683, circRNA_09923(down-regulation); and there are many cirRNA-miRNA-mRNA axis, such as, circRNA_23636-has-miR-6077-SLC2A1, circRNA_15734-hsa-miR-6893-5p-PFKFB3, circRNA_15734-hsa-miR-8485-VEGFA); circRNA_06551-hsa-miR-1915-3p- PFKFB3), circRNA_14668-hsa-miR-8485- VEGFA), circRNA_08683-hsa-miR-6728-5p-ENO2), circRNA_04497-hsa-miR-6808-5p-PFKFB3), circRNA_04497-hsa-miR-8485- VEGFA), circRNA_09923-hsa-miR-4755-3p- PFKFB3), circRNA_09923-hsa-miR-3155p-HK2), circRNA_09923-hsa-miR-3155a-HK2). Although the interaction between cirRNA-miRNA-mRNA axis has not been completely researched, however, the limitation of this article is that it is necessary to further confirm the role and mechanism of specific cirRNA-miRNA-mRNA axis in GIST imatinib resistance. We also speculate that differentially expressed circRNAs may play their biological functions in the process of IM resistance through interaction with miRNA-mRNA. In our next work, we will determine the expression of prediction circRNA, miRNA and mRNA in primary GIST and imatinib mesylate secondary resistance GIST, or with or without imatinib GIST cells; further verifying the cell activity, proliferation and apoptosis of these circRNAs against imatinib resistance in GIST cells following construction knockdown siRNA or overexpressed plasmids; use a dual-luciferase reporter assay to verify the relationship between circRNA and miRNA, or miRNA and mRNA, study the role of miRNA in the regulation of imatinib resistance in GIST cells by corresponding circRNA and further exploring the internal molecular mechanism of miRNA axis regulating imatinib resistance in GIST cells, at last, we also will demonstrate the role of cirRNA-miRNA-mRNA axis in regulating the imatinib resistance *in vivo*.

## CONCLUSION

This study serves as the first, to our knowledge, circRNAs sequencing and functional analysis in primary GIST and imatinib mesylate secondary resistance GIST. After IM failure, few therapeutic options remain, so it is urgent to identify the mechanism of drug resistance, HIF-1 seems to be crucial in future research.

## Figures and Tables

**Table 1:** General features of the circRNA sequencing data.

**Sample**	**Raw Reads**	**Raw Bases**	**Clean Reads**	**Clean Bases**	**Valid Bases**	**Q30**	**GC**
Sample_N1 Sample_N2 Sample_N3 Sample_C1 Sample_C2 Sample_C3 Sample_YC_1 Sample_YC_2 Sample_YC_3	84587074 91507150 91320298 84193988 82799646 85145886 84743526 83362440 82891684	10573384250 11438393750 11415037250 10524248500 10349955750 10643235750 10592940750 10420305000 10361460500	81651670 88469786 88647372 81676720 80243548 82621864 81602896 80906514 80622518	10201851969 11053620694 11076539842 10204694315 10025345565 10322885971 10186655571 10108276460 10073190258	96.48% 96.63% 97.03% 96.96% 96.86% 96.99% 96.16% 97.00% 97.21%	93.56% 93.73% 94.29% 94.20% 93.93% 94.11% 93.57% 94.37% 94.35%	55.50% 56.00% 57.00% 58.00% 58.00% 55.50% 57.00% 50.50% 55.50%

**Table 2:** Top 10 differentially expressed circRNAs in the three comparison groups.

**circRNA_id**	**circBase_id**	**log2FoldChange**	***p*-value**	**Adjusted *p*-value**	**Regulation**	**Host Genes**
**C-vs-N**						
circRNA_09533	hsa_circ_0006867	-8.62	9.25E-90	2.61E-85	Down	LRBA
circRNA_17427	hsa_circ_0018064	-inf	3.05E-64	4.30E-60	Down	SVIL
circRNA_20776	hsa_circ_0026782	-9.86	7.21E-53	6.78E-49	Down	ITGA7
circRNA_09530	-	-inf	2.72E-46	1.92E-42	Down	LRBA
circRNA_13055	hsa_circ_0079284	-5.41	2.84E-43	1.60E-39	Down	RNF216
circRNA_11884	hsa_circ_0004119	-5.40	1.63E-41	7.69E-38	Down	RAB23
circRNA_01991	hsa_circ_0005230	5.26	1.57E-35	6.32E-32	Up	DNM3
circRNA_13811	hsa_circ_0004365	-inf	7.44E-35	2.62E-31	Down	SEMA3C
circRNA_03576	hsa_circ_0000994	-4.03	2.99E-30	8.43E-27	Down	SLC8A1
circRNA_26953	hsa_circ_0000825	-5.70	2.70E-30	8.43E-27	Down	MTCL1
**C-vs-YC**						
circRNA_01991	hsa_circ_0005230	3.92	1.33E-19	2.49E-15	Up	DNM3
circRNA_03862	hsa_circ_0004435	-2.74	1.07E-12	6.67E-09	Down	FANCL
circRNA_15734	-	Inf	9.93E-13	6.67E-09	Up	-
circRNA_11269	hsa_circ_0003718	-4.00	6.83E-12	3.20E-08	Down	RANBP17
circRNA_02957	hsa_circ_0002922	-2.17	2.05E-11	7.68E-08	Down	ZNF124
circRNA_30179	-	-inf	3.18E-11	9.95E-08	Down	DIAPH2
circRNA_30540	hsa_circ_0009024	-inf	7.27E-11	1.95E-07	Down	-
circRNA_03489	hsa_circ_0000992	3.91	4.07E-10	9.55E-07	Up	PRKD3
circRNA_04497	-	-2.60	5.75E-10	1.20E-06	Down	DPP10
circRNA_25651	-	Inf	9.34E-10	1.75E-06	Up	ZC3H18
**YC-vs-N**						
circRNA_09533	hsa_circ_0006867	-8.15	1.75E-86	4.41E-82	Down	LRBA
circRNA_17427	hsa_circ_0018064	-inf	1.81E-67	2.28E-63	Down	SVIL
circRNA_13055	hsa_circ_0079284	-7.80	8.02E-60	6.75E-56	Down	RNF216
circRNA_04497	-	Inf	5.19E-58	3.27E-54	Up	DPP10
circRNA_09530	-	-inf	4.50E-47	2.27E-43	Down	LRBA
circRNA_11884	hsa_circ_0004119	-4.77	1.01E-39	4.26E-36	Down	RAB23
circRNA_20776	hsa_circ_0026782	-6.94	1.03E-33	3.70E-30	Down	ITGA7
circRNA_26999	hsa_circ_0008821	-inf	5.42E-32	1.71E-28	Down	RAB31
circRNA_13811	hsa_circ_0004365	-6.68	1.69E-29	4.74E-26	Down	SEMA3C
circRNA_18913	hsa_circ_0000277	6.50	1.12E-27	2.82E-24	Up	PDE3B

## Data Availability

We declare that all data supporting the conclusions of the study; all data generated or analyzed during this study are included in this published article and the data is available on reasonable request by the corresponding [J.Y.] author. The circular RNA sequencing analysis and conducted by OE Biotech Co., Ltd, the data analysis, Figures/Schemes were from authors group team.

## LIST OF ABBREVIATIONS

GIST: Gastrointestinal Stromal Tumor
KEGG: Kyoto Encyclopedia of Genes and Genomes
GO: Gene Ontology
RIN: RNA Integrity Number
IM: Imatinib
EMT: Epithelial-Mesenchymal Transition
NSCLC: Non-Small Cell Lung Cancer
